# Enablers and barriers for implementing high-quality hypertension care in a rural primary care setting in Nigeria: perspectives of primary care staff and health insurance managers

**DOI:** 10.3402/gha.v9.29041

**Published:** 2016-02-12

**Authors:** Aina O. Odusola, Karien Stronks, Marleen E. Hendriks, Constance Schultsz, Tanimola Akande, Akin Osibogun, Henk van Weert, Joke A. Haafkens

**Affiliations:** 1 Department of Global Health, Academic Medical Center, University of Amsterdam and Amsterdam Institute for Global Health and Development, Amsterdam, The Netherlands; 2 Department of Public Health, Academic Medical Center, University of Amsterdam, Amsterdam, The Netherlands; 3 Department of Epidemiology and Community Health, University of Ilorin Teaching Hospital, Ilorin, Nigeria; 4 Department of Community Health, Lagos University Teaching Hospital, Lagos, Nigeria; 5 Department of General Practice, Academic Medical Center, University of Amsterdam, Amsterdam, The Netherlands

**Keywords:** hypertension, primary care, community-based health insurance, sub-Saharan Africa, stakeholder perspectives, qualitative study

## Abstract

**Background:**

Hypertension is a highly prevalent risk factor for cardiovascular diseases in sub-Saharan Africa (SSA) that can be modified through timely and long-term treatment in primary care.

**Objective:**

We explored perspectives of primary care staff and health insurance managers on enablers and barriers for implementing high-quality hypertension care, in the context of a community-based health insurance programme in rural Nigeria.

**Design:**

Qualitative study using semi-structured individual interviews with primary care staff (*n* = 11) and health insurance managers (*n*=4). Data were analysed using standard qualitative techniques.

**Results:**

Both stakeholder groups perceived health insurance as an important facilitator for implementing high-quality hypertension care because it covered costs of care for patients and provided essential resources and incentives to clinics: guidelines, staff training, medications, and diagnostic equipment. Perceived inhibitors included the following: high staff workload; administrative challenges at facilities; discordance between healthcare provider and insurer on how health insurance and provider payment methods work; and insufficient fit between some guideline recommendations and tools for patient education and characteristics/needs of the local patient population. Perceived strategies to address inhibitors included the following: task-shifting; adequate provider payment benchmarking; good provider–insurer relationships; automated administration systems; and tailoring guidelines/patient education.

**Conclusions:**

By providing insights into perspectives of primary care providers and health insurance managers, this study offers information on potential strategies for implementing high-quality hypertension care for insured patients in SSA.

## Introduction

Hypertension is a leading risk factor for cardiovascular disease (CVD) and premature mortality (1), with a global prevalence of 22% in people aged 18 years and older in 2014 (2). The highest rates were found in sub-Saharan Africa (SSA) (2). In Nigeria, the estimated overall hypertension prevalence is 28.9% (3). Reduction of blood pressure (BP) through timely and sustained treatment reduces mortality resulting from CVD (4–6). Evidence-based guidelines for the treatment of hypertension in Africa have been available since 2002 (7–9), but treatment rates are still alarmingly low (10, 11). This is a major contributor to the growing CVD burden in SSA (1).

Many of the reasons for the under-treatment of hypertension in SSA countries are related to their weak health systems (12), including a lack of facilities, trained staff, essential drugs and organisational capacity for the delivery of chronic care (13–15) and the non-affordability of care for patients (15, 16). According to the World Health Organization (WHO), CVD prevention in local communities is one of the ‘best bets’ for low- and middle-income countries (LMICs) to curb the growing burden of CVD (17, 18). In response to this information, in a number of SSA countries new programmes are being developed to strengthen CVD prevention and hypertension care in urban and rural primary care settings (19, 20). The implementation of such programmes can be conceived of as an innovation in healthcare practice.

Implementation science research has demonstrated that a range of context-specific factors may either limit or enable the uptake of innovations in practice (21–26). Collectively, these factors are known as *determinants of practice* (26). Systematic review evidence suggests that innovation in healthcare is more likely to succeed if implementation strategies are tailored to context-specific determinants of practice (27, 28). To date, research on factors that may prevent or facilitate the implementation of high-quality hypertension care has mainly been conducted in healthcare settings in high income countries (27, 29, 30). Given the context-specific character of determinants of practice, comparable studies are urgently needed in LMICs as well.

The study reported in this paper is part of a larger research project that aimed to investigate how health insurance can be used for CVD prevention in low-resource communities in SSA (19, 31). This research project was carried out in the context of a community-based health insurance (CBHI) programme in rural Kwara State, Nigeria (32–34). Associated studies from our research group reported on patient perspectives on factors enabling or inhibiting adherence to antihypertensive treatment (35), the burden of CVD risk factors in the communities eligible for the CBHI programme (36), the effect of health insurance on BP in individuals with hypertension (34), the effect of patient education on insured patients who were treated for hypertension (37), and the operational feasibility of implementing CVD prevention guidelines in a primary care setting (38).

With the objective of contributing to a better understanding of strategies that are needed to facilitate the uptake of high-quality hypertension care in primary care settings in SSA, the aim of the present study was to explore perspectives of insurance managers and primary care staff on factors that might inhibit or facilitate the implementation of high-quality hypertension care in practice. As financers and providers of care, respectively, both groups were important stakeholders in the quality improvement of CVD prevention and hypertension care in rural Kwara.

## Methods

This study was conducted among insurance managers of the Kwara State Health Insurance (KSHI) and primary care staff of a KSHI clinic. We employed a qualitative design and semi-structured individual interviews (39) to explore stakeholders’ perspectives. We followed the consolidated criteria for reporting qualitative research (COREQ) checklist for reporting the results (40).

### Context and setting

#### Kwara

Located in western Nigeria, Kwara State is the fourth poorest state of the country (32). The majority of the population lives in rural areas (67% of 2,748,400; data from 2011 census). The programme areas included in KSHI are rural, low-income farming communities. Yoruba (67.8%) and Nupe (9.9%) are the two largest ethnic groups in these areas, Islam and Christianity the main religions, and trading and farming the main occupations. In 2009, 20% of the population lived below the poverty line of 2 US dollars (USD) per day; 40% and 25% had completed primary school and senior secondary school, respectively; and 44.3% were illiterate (34).

#### KSHI

KSHI is a voluntary CBHI programme that commenced in 2007 in three regions in Kwara State, under the name Hygeia Community Health Plan (32). The programme provides coverage for primary and limited secondary care for enrolees and supports the quality improvement of care in participating clinics. KSHI is financed by the Health Insurance Fund (HIF) and the Kwara State government. It is implemented in the programme areas by an international development organisation, the PharmAccess Group, and the Nigerian insurance company Hygeia Health Maintenance Organisation. Beneficiaries are enrolled individually on an annual basis and pay a share of 12% of the insurance premium. Prior to 2009, HIF subsidised the rest of the premium through a grant from the Dutch government. Since then, the Kwara State government has also supported KSHI. In 2014, the state government subsidised 60% of the premium and pledged to take over the full funding of KSHI and its scale-up in the state in the near future. (For a detailed description of KSHI, see Supplementary file – KSHI.)

As part of its quality improvement programme, the insurance programme introduced the WHO and other international guidelines for hypertension and CVD prevention care (4, 41) in a number of contracted clinics in 2010. It provided the clinics with new equipment, organisational support, and staff training to facilitate implementation. The international SafeCare programme (42) supports quality improvement programmes for KSHI and monitors the results.

Our study took place in 2010, immediately after the guidelines were introduced in KSHI clinics. In line with recommendations from previous studies on implementation of quality improvement in healthcare (23), we were particularly interested to learn what stakeholders perceived as inhibitors or facilitators of care at the very start of this innovative programme. In 2010, 32% of the targeted individuals in KSHI's programme area had enrolled in the insurance plan (*n*=24,763) (43). Enrolees paid 7% of the annual premium themselves: 300 Nigerian naira (NGN) or 2 USD (32). Provider clinics received a capitation fee for all patients who were registered at KSHI, irrespective of their care utilisation. In addition, they received a fixed monthly extra fee of 2,000 NGN (USD 12.50) for each hypertension patient who visited their clinic in a given month (43). The combination of revenue from capitation fees and monthly fees for consulting hypertensive patients was meant to cover all treatment costs for out-patient hypertension care, including consultations, diagnostic tests, and drug treatments.

The primary care clinic where we recruited healthcare staff had about 400 insured patients who were treated for hypertension and 24 staff members at the time of the study. Staff members included three primary care physicians, one of whom was a medical director and two of whom were residents in training; 10 general staff nurses and midwives; three pharmacy technicians; four laboratory technicians; and four health records and patient administration staff. To protect the anonymity of the study participants, we will not provide further detail on this clinic.

### Sampling

We used purposive sampling (44, 45) to recruit clinic and health insurance staff. Our purpose was to interview informants with a variety of professional backgrounds who could share their experience in hypertension care with us. Therefore, we decided to recruit clinic staff with at least 1 year of experience in clinical or administrative aspects of hypertension care and insurance managers who had liaised with contracted clinics about standards of CVD prevention care for at least 1 year. In qualitative interview studies, data saturation is a criterion to determine sample size (46). This means that the number of respondents is sufficient if interviews with new respondents do not yield new themes. In this study our possibilities to determine sample size based on data saturation were, however, restricted by practical circumstances. We were only able to locate 11 clinic staff and 4 insurance company staff who met our inclusion criteria, all of whom agreed to be interviewed. As intended, this group included a good variety of professionals (see ‘Results’ section). With the 15 interviews we were nonetheless able to achieve data saturation. Indeed, as a study of the sample sizes used in qualitative studies has shown, sample sizes of 15–17 respondents are generally sufficient to achieve data saturation (46).

### Data collection

Data were collected through in-depth individual interviews (39), guided by a topic list. Our topic guide was based on a previous study on implementation of an innovative patient education programme in a primary care setting in Europe (47) and field experience in quality-of-care monitoring programmes in Nigeria (42). Semi-structured open-ended questions were used to explore the following topics: 1) the current way in which hypertension care is provided; 2) barriers that can make it difficult to provide high-quality hypertension care; and 3) enablers that can facilitate the provision of (consistent) high-quality hypertension care. For each topic various probes were used, which were made relevant for the functions of each participating respondent group if needed (see Supplementary file – Topic list). After acquiring informed consent, the researcher (AOO) conducted the interviews in English, the lingua franca used by healthcare and insurance staff in the region. Using a voice recorder, all interviews were held at the respondents’ own workplaces. After each interview, the researcher immediately wrote field notes describing the context of the interview, transcribed the interviews with the help of assistants, and wrote a memo summarising the main themes that were addressed by the respondent. After reviewing all 15 interview transcripts, we (AOO, JAH) decided to carry out follow-up interviews with three respondents to further explore and check some of the themes that had remained unclear in the previous interviews. Respondent validation is a common exercise in qualitative research to confirm or refute interpretations and to refine data (45). The first set of interviews with all 15 respondents lasted about 90 min each. The follow-up interviews with three respondents were held about 6 weeks after the initial interviews and lasted 25 min each.

### Data analysis

We used the qualitative data management software MAX Qualitative Data Analysis (MAXQDA) to support consistent analysis, processing, ordering, and comparison of the data (48). As a first step, all interview transcripts were read by AOO and sections containing information about respondents’ perceptions on inhibitors and enablers of quality hypertension care were selected. As a second step, all selected sections of the interview transcripts were analysed inductively for thematic content, using coding techniques and procedures described by Strauss et al. (49). Briefly, the process of inductive data analysis began with the assignment of a series of open codes to the interview transcripts, which were then grouped into clusters (concepts) in order to make them more workable. From these concepts, broader categories and subcategories were generated in several rounds through a process of constant comparison or verification. Finally, for each stakeholder group (healthcare staff and insurance managers) the results of these multilevel coding procedures were summarised into matrices in which perceptions on inhibitors and facilitators were summarised, in terms of categories, subcategories, and concepts (for an example, see Supplementary file – Matrix).

As a third step of the analysis we applied a deductive method and grouped the inductively created (sub-) categories under broader themes. To this end we used a comprehensive theory- and research-based conceptual framework that was published by members of the European Tailored Implementation for Chronic Diseases (TICD) network in 2013 to guide research on the implementation of healthcare improvements in healthcare practice (26). The TICD framework describes factors (determinants of practice) that may inhibit or enable the uptake of innovations in primary care practices. It comprises seven broad domains (guideline factors; health professional factors; patient factors; professional interactions; incentives and resources; capacity for organisational change; and social, political, and legal factors), each of which is divided into a range of subdomains. We compared the inductively created categories that had emerged from our analysis of the interviews with each of the (sub-) domains in this framework. After reflection (50), there was a common conclusion among members of the research team that many of these categories were related to the TICD domain *resources and incentives* and its subdomains. Therefore, we used this TICD domain as an overarching thematic framework to further categorise and describe our inductive results (Tables 2 and 3).

The first two steps of the analysis took place in the first 18 months following data collection. The last step took place 2 years later.

#### Trustworthiness

To ensure a rigorous research process and credibility and trustworthiness of the findings, the following steps were taken during this study:

1. After the first 15 interviews were held, two researchers (AOO, JAH) read the transcripts and the memos and established together what additional information would be needed from respondents to verify and substantiate the initial themes that emerged from the data (45).
2. AOO selected the interview fragments that were analysed in this study and performed the initial open coding procedure, which resulted in a codebook. JAH reviewed this codebook and checked whether the codes were workable and reflected the underlying content.
3. AOO and JAH categorised the codes into broader concepts independently in three rounds, compared results, discussed whether the broader concepts reflected the codes and the underlying data, and reached agreement about the final list of concepts through discussion.
4. The next step of coding, where concepts were organised under major categories and subcategories, was performed by AOO and JAH separately in several rounds. The thematic matrixes that emerged from these exercises were discussed and differences in opinion were resolved through consensus. The final matrices were discussed in the core research group (KS, HW, CS) with the help of AO.
5. The deductive analysis was performed by AOO, KS, and JAH. They independently compared the inductively created categories with the domains and subdomains in the TICD framework. By using a reflective process (50) that is common in qualitative research, they came to an agreement that most categories referred to and could be grouped within subdomains of the TICD domain *resources and incentives*.
6. Final results were discussed with all other members of the research team, and interpretations or results that could not be justified by the data were reconsidered and revised.

## Results

All invited participants agreed to be interviewed (*n*=15). At the clinic, we interviewed two physicians (a medical director and a resident in training), two nurses working in the hypertension programme, one member of the pharmacy staff, two laboratory staff who conducted CVD-related lab tests, and four administrative staff, who were responsible for patient records and hospital management administration. We also interviewed four health insurance managers from KSHI, all medical doctors. Most participants were middle-aged (20–40 years) and two-thirds were male (Table 1). Most primary care staff had worked less than 5 years at the clinic. Insurance managers had worked between 1 and 3.5 years for the KSHI programme.

**Table 1:** Background characteristics of participants (*n*=15)

Characteristics	*n*
Age group (years)	
21–30 years	8
31–40 years	6
51–60 years	1
Gender	
Male	10
Female	5
Stakeholder group	
Clinic	11
Insurance company	4
Working experience of clinic staff	
1–5 years	9
6–10 years	1
>10 years	1
KSHI managers' work experience	
0–2 years	2
>2–4 years	2

KSHI, Kwara State Health Insurance.

### Perceived enablers and barriers for implementing high-quality hypertension care: clinic staff

The inductive analysis of the interviews with clinic staff yielded 12 major categories referring to enablers or inhibitors for providing high-quality hypertension care. Most categories dealt with resources/conditions that are needed for the delivery of care. The deductive analysis found that the 12 categories could be grouped under six overarching themes, all of which refer to subdomains of the aforementioned domain *incentives and resources* of the TICD framework: 1) necessary resources; 2) financial incentives and disincentives; 3) non-financial incentives and disincentives; 4) information systems; 5) quality assurance and patient safety systems; and 6) continuing education system. The results are summarised in Table 2.

**Table 2:** Overview of healthcare providers’ perspectives on enablers and barriers for implementing high-quality hypertension care in a rural primary care facility by theme and category^a^^,^^b^^,^^c^

Theme/category	Factors enabling high-quality hypertension care	Factors inhibiting high-quality hypertension care
1. Necessary resources		
1.1 Health insurance	Subsidised health insurance is vital for providing standardised hypertension care for low-income patients. (R1, R2)	Standardised hypertension care is not sustainable if subsidy for insurance premiums is no longer available. (R1, R2)
	Participation in health insurance makes upgrading of quality of hypertension care to desirable levels possible for low-resource facilities. (R2)	Costs of diagnostic services and CVD preventive screening are not fully covered by insurance. (R2) Implementation of clinic upgrades depends on availability of trained personnel. (R2)
1.2 Guidelines, protocols, tools	Availability of treatment guidelines, protocols, and SOPs. (R1, R2)	Certain dictates of international treatment guidelines are not applicable in some specific local contexts or cannot be implemented due to resource constraints. (R1, R2) Lack of interpreters/language tools for professional–patient communication can hinder adherence to guideline-based care. (R1, R2) Lack of tools for ‘culturally tailored’ patient education can hinder patient adherence to care. (R1, R2, R3)
1.3 Human resources	Availability of trained personnel to diagnose, investigate, treat, and educate patients that present with CVD risk factors. (R1, R2, R3, R4, R5, R6)	Clinic's current personnel shortages (doctors, nurses, pharmacy staff, and lab staff) hinder vital aspects of care: treatment, patient education, and investigations. (R1, R2, R3, R4, R6)
1.4 Equipment/supplies	Availability of sufficient diagnostic equipment, consumables, and medications promotes care. (R2, R4, R9) Equipment maintenance plan. (R2, R4, R9, R5) Alert systems to forestall (avoidable) shortages of materials. (R7, R5)	Inadequate availability of vital diagnostic equipment; shortages in supply of lab consumables and drugs. (R2, R4, R9) Poor equipment maintenance culture. (R2, R5, R6)
1.5 Health records and patient follow-up	Implement health records system that enables identification and follow-up of patients. (R1, R2, R8, R9, R10, R11) Contact tracing through home visits/phone calls. (R8, R9, R11)	Poorly implemented follow-up appointment system resulting in treatment non-adherence and poor patient outcomes. (R2, R8, R9, R10, R11) Some patients have no telephone or fixed address. (R8, R9, R10)
2. Financial incentives and disincentives		
2.1 Insurance claims management system Remuneration system	Adequate and timely compensation by the insurance company for all care services duly rendered by the provider. (R2) In addition to capitation payments, ‘fee for service’ remuneration would enable screening and early preventive treatment where needed. (R2)	Recurrent delays and inconsistencies in settlements of verifiable claims. (R2) Reimbursement of CVD prevention through current remuneration system demotivates providers and underfunds certain aspects of care. (R2)
2.2 Benefits package of rural workers	Enhanced salary/benefits package will motivate and retain rural healthcare personnel. (R1, R3, R5, R6, R9, R10, R11)	Poor remuneration and poor living conditions dampen morale of rural healthcare workers. (R5, R1)
3. Non-financial incentives and disincentives		
3.1 Provider–insurer working relationship	Constructive dialoguing between provider and insurer. (R2)	Insufficient communication with insurer can hinder administrative processes and the quality of CVD preventive care. (R2)
4. Information systems		
4.1 Information technology systems	Implement electronic health management information system to facilitate and optimise administration of care. (R1, R2)	Dysfunctional computers/Internet connections negatively impact quality and output of care. (R2)
5. Quality assurance and patient safety systems		
5.1 Reliability of laboratory results	Implement internal and external quality control processes for laboratory investigations to assure quality and reliability of laboratory results. (R5, R6)	Lack of credible quality assurance system creates doubt in the laboratory results used for monitoring progress of care. (R5)
5.2 Reliability of vital consumables	Ensure potency of vital consumables using certified, credible suppliers and a ‘near expiry’ alert system for drugs/laboratory consumables. (R2, R4)	Uncertainty about potency of vital consumables used for CVD care may lead to poor patient outcomes. (R2)
6. Continuing education system		
6.1 Training for staff	Consistent skills update trainings for healthcare workers to promote quality of CVD prevention care. (R1, R2, R3, R4, R5, R6)	Lack of institutionalised system for continuous knowledge renewal through refresher trainings on CVD prevention care can hinder the quality of care. (R1, R5, R6)

CVD, cardiovascular diseases; SOPs, standard operating procedures.

a *Theme* refers to specific subdomains grouped under the TICD domain *resources and incentives* (26).

b *Category* refers to inductively identified categories in this study.

c *R* refers to ID numbers given to respondents.

#### Theme 1: availability of necessary resources

1.1 The clinic staff described the availability of *affordable health insurance* as one of the most important resources that had made it possible to provide high-quality hypertension care to the local, mostly poor, population:

This health insurance has been the main driver that enabled people to now access quality care which they did not have before; by paying just 200/300 naira a whole year, and the insurance pays the rest for them … I used to think before now that hypertension and diabetes were not common … In fact, most of our adult consultations now are due to hypertension apart from the obstetric and gynaecologic care. (R2)

The insured patients are better compliant with visits, drugs, and advices and are better controlled. This has a lot to do with the fact that barriers to access care have been removed by the insurance. (R1)

Yet, some clinic staff also viewed ‘the dependency of the poor’ on ‘subsidised’ health insurance as a potential threat to the sustainability of quality hypertension care:

I will be very honest with you; if the insurance subsidy stops, the program will simply collapse unless there is an alternative subsidy. (R2)

Secondly, clinic staff perceived the support from the insurance company for equipment upgrades and the development of quality improvement plans at their clinic also as an incentive for improving hypertension care. Nevertheless, despite this support, some staff members still experienced certain inhibitors to the delivery of quality hypertension care. They pointed out that high-quality care requires regular diagnostic and screening tests to prevent or detect CVD (e.g. lipid profile, electrocardiogram, electrolytes, urea, creatinine, microalbuminuria, glycosylated haemoglobin). In the perception of some clinic staff, the costs of these services were not fully covered by the insurance reimbursement system. In addition, it was noted that the actual implementation of clinic upgrades was challenged by the limited availability of trained personnel:

Since KSHI program came and upgraded our laboratory for us, we can do many additional tests … But the success of doing all CVD related tests […] depends on availability of qualified personnel. (R2)

1.2 The *guidelines and protocols* that were introduced by KSHI as part of its quality assurance programme were seen as a relevant incentive for improving quality of hypertension care in the clinic. However, some clinic staff noted that international (clinical) guidelines do not always provide sufficient information to make them applicable in local contexts:

If we use the [WHO] guideline [which recommends three monthly clinic visits] many patients would end up not using drugs appropriately; they will mismanage drug stocks because of longer times between appointments; storage becomes difficult also … For now I think the once-monthly arrangement should be appropriate for the literacy level of the patients we see here. (R1)

In addition to guidelines, the clinic staff emphasised that other instruments are also needed to encourage patients’ adherence to guideline-based care. They emphasised the need for tools that allow for good communication between providers and patients, particularly in a context where the local population speaks many different languages:

Language barriers with patients are also sometimes a limitation during consultation in a setting such as ours (where people speak different languages) because even the interpreter (if you have one) can sometimes inadvertently give wrong information. (R1)

They also felt there was a need for educational tools that make it possible to address the specific cultural and local understandings of health and healthy behaviour among the patient population in the area.

1.3 All clinical staff perceived the consistent availability of qualified healthcare personnel (*human resources*) as a vital resource for implementing high-quality hypertension care and education. Since the introduction of the health insurance programme, the number of patients who sought hypertension care at the clinic had increased significantly. The increased patient load had brought the issue of acute personnel shortages to light, which was viewed as a barrier to quality care.

I see on average 45 hypertensive patients on hypertension clinic days (held every Friday). This number is too much. […] Consulting time is 10 to 15 minutes, but the ideal time for our level of development should be 30–40 minutes. Especially if the patient is coming for the first time we spend almost an hour including educating them. (R1)

1.4 Since the introduction of the KSHI programme, *diagnostic equipment*, *drugs*, *and laboratory consumables* for the provision of quality care were more readily available. Clinic staff emphasised, however, that the facility also needed to take additional measures to ensure the proper use of these materials, such as the implementation of an ‘equipment maintenance plan’ to address ‘the poor equipment maintenance culture’ and an ‘alert system’ to forestall supply shortages (see also Subsection 5.2).

1.5 Both medical and administrative officers felt that adequate *health records and patient follow-up* were essential for quality care:

We have recently improved our (manual) documentation system. The folders have columns and rows for every patient, so we don't miss things, and we document drugs, treatment, and follow-up appointment. (R1)

Linked to this, the clinic was setting up a patient recall/reminder system to help track patients and identify those who don't show up at appointments, so as to encourage adherence:

We have a recording book for patients’ visits and appointments. We know if patients don't turn up for appointment. We then make phone calls to recall them. If necessary, we use their contact addresses to go and search physically for them in their villages. (R9)

However, the administrative officers noted the limitations of this contact tracing system, as it only works if patients have traceable addresses.

#### Theme 2: financial incentives and disincentives

2.1 In the interviews, the clinical staff pointed at problems in the management of health insurance claims, such as late payments. In addition, they felt that the combination of capitation payments and a monthly fixed additional fee per patient for hypertension care utilisation was not always sufficient to cover all the costs of care that are recommended by hypertension guidelines, for example specific laboratory investigations.

2.2 Most healthcare staff felt an enhanced *salary and welfare package* was essential to attract, retain, and motivate *healthcare personnel in rural communities* and to ensure high-quality care:

The current salary package is a limitation to us and one wouldn't mind seeking government employment [elsewhere] in order to get better package than now; the hospital is trying its best though, but will need assistance on this. (R6)

#### Theme 3: non-financial incentives and disincentives

3.1 The clinic staff felt that constructive dialoguing and fostering of a *good provider–insurer working relationship* was essential in order to prevent and remove prevailing organisational and administrative obstacles to quality care:

One major challenge now is that we have not really had time to sit down as stakeholders to dialogue over practical realities of implementing some of the program recommendations in our own setting here. There is [a] communication gap. We need to have a stakeholder session for others to also see what we experience in implementing this program. (R2)

#### Theme 4: information systems

4.1 As typical for facilities in remote areas, the *information and communication technology system* was also poor at the clinic where our respondents worked. Computers were out-dated and Internet connections often dysfunctional. The clinic staff strongly believed that the improvement of hardware and management software would be an important incentive for better care and management:

For over a year now, the internet service has not worked. This contributes to our own financial loss because we do many things that cannot immediately be mailed out or communicated to the insurance company and this has affected our revenue negatively while our services and wage bills are rising. (R2)

#### Theme 5: quality assurance and patient safety systems

5.1 Efficient internal and external quality control systems were considered essential to ensure quality of care, such as in the case of *laboratory results*:

We have recently commenced internal quality control for our spectrophotometer and if the control is out of range during a quality control test, the result will not be issued out. […] Quality control determines the quality of our laboratory results. (R5)

5.2 The availability of a protocol to ensure that vital supplies like *drugs and laboratory consumables are potent and unexpired* when used was also viewed as an essential condition for quality CVD prevention care. Not oblivious of the potential ease with which fake drugs and medical supplies can be acquired in Nigeria, health professionals mentioned that reliable ‘test’ and ‘near expiry alert’ systems and quality-certification of suppliers of medical consumables was important.

#### Theme 6: continuing education system

6.1 Regular *professional development for staff* was perceived as pivotal for implementing high-quality hypertension and CVD prevention care. KSHI's training programme was considered very relevant and the clinic staff would appreciate opportunities for further specialisation in CVD care:

If given the opportunity, I [would] be very happy to specialise as a cardiovascular nurse as this [would] also help to reduce the mortality and morbidity associated with these diseases. It is not good to stay without increasing knowledge. (R3)

### Perceived enablers and barriers for implementing high-quality hypertension care: health insurance staff

Table 3 provides an overview of themes and categories that emerged from our analysis of interviews with health insurance staff. Many of them are similar to those found in the interviews with the clinic staff. However, they referred to these themes from a health insurers’ perspective: *I see myself as a catalyst in ensuring quality care from providers to the enrolees* (IR1).

**Table 3:** Overview of health insurance managers’ perspectives on enablers and barriers for implementing high-quality hypertension care in a rural primary care facility, by theme and category^a^^,^^b^^,^^c^

Theme/category	Factors enabling high-quality hypertension care	Factors inhibiting high-quality hypertension care
1. Necessary resources		
1.1 Health insurance	Health insurance makes CVD prevention and hypertension management affordable for enrolees. (IR1, IR2, IR3, IR4) If subsidy from third parties stops, resources in community will be found to sustain the programme. (IR1, IR2, IR4)	Patients spend more than the annual premium on transport to clinic. (IR2)
	Proactive care approach by insurance benefits population health and insurance. (IR1, IR2, IR3, IR4)	
	The insurance programme's quality improvement and education policy facilitates delivery of standardised CVD prevention care in contracted hospitals. (IR1, IR2, IR3)	Resource constraints experienced by healthcare providers hinder implementation of recommended improvements. (IR3, IR2)
1.2 Guideline and protocols	Insurance company uses guidelines to monitor and ensure high-quality care. (IR1, IR2, IR4)	Inconsistent and inadequate use of guidelines by healthcare professionals hinders care. (IR2, IR4)
1.3 Equipment and supplies	Clinic upgrades and monitoring activities substantially minimised shortages of essential drugs, diagnostic tools, and materials. (IR3, IR2)	Some providers lack capacity/will to fund complementary acquisition of diagnostic tools and materials. (IR4, IR2)
2. Financial incentives and disincentives		
2.1 Insurance claims management system	Quick claim settlement motivates providers. (IR1, IR2, IR3, IR4)	Claim verification process is time-consuming and intensive for insurance company. (IR2, IR1, IR4)
2.2 Remuneration	A fixed extra fee on top of regular monthly capitation fee per patient promotes quality of CVD preventive care. (IR2)	Providers want capitation and ‘fee for service’ payments. (IR2, IR1)
2.3 Benefits package of rural workers	Government and providers must improve welfare of rural health workers. (IR1)	Rural-based providers have no funds to improve welfare of health workers unilaterally. (IR1)
3. Non-financial incentives and disincentives		
3.1 Provider–insurer relationship	M&E essential to ensure that hypertension/CVD preventive care is delivered according to standard. (IR1, IR2,IR2, IR4) Feedback, training, and teamwork will minimise credibility issues arising from monitoring of quality of care. (IR1)	Some providers see M&E as a threat. (IR1, IR3)
4. Information systems		
4.1 Information technology systems	A functional ICT system will facilitate efficient administration and promote quality of care. (IR1, IR2, IR3, IR4)	Dysfunctional information technology infrastructure hinders provider–insurer communication, leads to inefficient administration, and diminishes quality of care. (IR1, IR2, IR3, IR4)
5. Quality assurance and patient safety systems		
5.1 Monitoring all aspects of treatment including patient satisfaction	Patient file checks to verify drugs, lifestyle advice, other treatment, BP outcomes, pharmacy stock, and quality reviews. (IR1, IR3, IR4) Laboratory checks. Certification of suppliers for drugs, laboratory reagents, and other materials. (IR1, IR2, IR2, IR4) Mystery shopping and surveys to investigate patient satisfaction. (IR1, IR2, IR4)	
6. Continuing professional education system		
6.1 Training for providers	Continuous skills improvement and update trainings made available for health professionals. (IR1, IR2, IR4)	High attrition of rural health workers means limited benefits of training to patients. (IR4)

M&E, monitoring and evaluation; ICT, information communication technology; BP, blood pressure.

a *Theme* refers to subdomains grouped under the TICD domain *resources and incentives* (26).

b *Category* refers to inductively identified categories in interviews with health insurance managers in this study.

c IR1, IR2, IR3, and IR4 refer to ID numbers given to respondents.

#### Theme 1: availability of necessary resources

1.1 Health insurance staff stated that *health insurance* and ‘risk pooling’ are essential to improve the quality of life of the local population. They argued that people with chronic conditions, like hypertension, are the greatest beneficiaries of health insurance schemes:

Health insurance benefits chronically ill patients with hypertension and diabetes since it increases access to care and alleviates costs of managing these conditions. (IR4)

One informant noted, however, that additional (travel) costs may still hamper access to care for patients. Overall, managers were more optimistic than healthcare providers about the financial sustainability of the insurance programme:

Even if external funding stops later, as expected, people will have experienced the benefits of the (insurance) program and will be willing to pay fully by themselves, thus driving feasibility and sustainability. (IR2)

All interviewees emphasised that it is the insurance company's policy to promote a ‘proactive’ approach to CVD management, which focuses in the first place on prevention:

Proactive management is currently not well done in provider settings. Ideally it should involve the healthcare provider marshalling all resources at its disposal to treat, inform, and educate patients in attempts to prevent CVD. (IR4)

One important consideration for promoting a proactive approach is that it benefits individual patients and the population as a whole:

When CVD is prevented in a patient you promote good quality of life in him, make him more useful to his family and the society, reduce costs [to] the insurance company, reduce the workload in [the] provider hospital, and for the country at large you reduce [the] morbidity and mortality burden. (IR3)

The interviewees also viewed the insurance company's *quality improvement programme* as an important resource for the improvement of hypertension care in contracted clinics. However, they observed that financial constraints at local clinics could still hinder implementation:

Some providers find it difficult to implement the upgrade plan that we recommended to them, because of lack of funds on their part. (IR4)

More specifically, personnel shortages and the concomitant long waiting time for patients were seen as one of the main challenges to delivery of high-quality care in rural primary care settings. Some informants feared, however, that ‘quality upgrades’ that the insurance company had provided to clinics could make providers ‘overly dependent’ on external support, failing to implement (complementary) upgrade plans of their own.

1.2 Like the clinic staff, health insurance staff saw the introduction of *international guidelines* as one of the key instruments that could help improve the quality of care by providers and the monitoring of care by the insurance company.

1.3 According to the health insurance staff, the KSHI programme's monitoring activities support clinics to minimise shortages and maximise the quality of vital *equipment*, *supplies*, *and drugs* needed for high-quality care.

#### Theme 2: financial incentives and disincentives

2.1 Even though insurance managers agreed with providers that the *prompt settlement of claims* would motivate quality care, they emphasised that for an insurance company the proper verification of claims is crucial and time-consuming:

The process of verifying claims and removing errors takes quite some time. We visit hospitals, sometimes to audit their case files and see whether the claimed care was actually delivered. (IR3)

2.2 The health insurance staff agreed with the perception of the clinic staff that *adequate provider remuneration* acts as an incentive for quality care. They differed, however, in their perception of what provider payment system is feasible in the context of a health insurance programme. They pointed out that the KSHI programme offers a fixed ‘extra’ fee on top of the ‘standard capitation fee’ to compensate providers for the financial burden of chronic CVD prevention and hypertension care:

Our company's administrative policy compensates chronic disease care under capitation payments. However, for CVD prevention care we offer them an additional NGN 2000 per month for each hypertension patient who utilizes care that month. (IR2)

They pointed out that this administrative policy is built on the view that the current remuneration system is developed with a focus on what the patient in a low-resource setting truly needs, namely basic preventive care to forestall serious illness and a low premium for health insurance.

2.3 Most managers recognised that the *poor benefits package* for rural health workers could negatively impact care:

Rapid employee turnover and low moral from the staff because of issues related to employee well-being are some of the limitations encountered by providers. (IR1)

#### Theme 3: non-financial incentives and disincentives

3.1 In the perspective of health insurance staff, monitoring and evaluation of contracted provider hospitals is a vital strategy for ensuring high-quality (hypertension) care. However, they observed that this could disrupt a good *provider–insurer relationship*, whereas some providers see monitoring activities as a threat. In order to improve ‘teamwork’ between stakeholders, insurance managers said they were using several strategies, such as the provision of feedback to clinics on how they are improving, staff training, and the establishment of cordial relations with hospital directors:

We give feedback to the hospital and dialogue with the staff and try to find amicable solutions to problems. (IR2)

#### Theme 4: information systems

4.1 Like the clinic staff, health insurance staff perceived the availability of a *functional and effective information technology system* as being critical for an efficient administration and implementation of the CVD prevention programme.

#### Theme 5: quality assurance and patient safety systems

5.1 The KSHI programme conducts regular pharmacy and laboratory checks in contracted clinics and ensures that suppliers of drugs and other materials are certified. In the eyes of health insurance staff, external quality assessment is not only necessary for materials and consumables but also for *real aspects of treatment*:

We use [patients’] case file data to verify prescribed/dispensed drugs, behavioural advice, improvements in BP, other disease data, and target organ screening. (IR3)

Moreover, they highlighted the importance of monitoring patients’ *experiences with care:*

We do mystery shopping once in a while, and carry out regular patient satisfaction surveys on providers. (IR3)

#### Theme 6: continuous professional education

6.1 Like the clinic staff, health insurance staff attributed great value to the implementation of a *professional development* programme for the contracted clinics, even though they observed that clinics may not get lasting benefits from staff training, due to the high turnover of health workers.

## Discussion

This study provides insight into the perspectives of primary healthcare staff and health insurance staff on enablers and inhibitors for implementing high-quality care for insured hypertensive patients in rural Nigeria. The study was conducted in the context of a CBHI programme that targets low-income groups and offers quality improvement programmes to participating clinics.

From the perspectives of our informants a range of resources and incentives are needed to enable the delivery of high-quality hypertension care in their region: affordable care for patients, treatment guidelines, tools for patient education, sufficiently trained staff, diagnostic equipment and medication, adequate patient and hospital management systems, quality assurance mechanisms, and adequate reimbursement of provider services. Studies from other LMICs (12, 13, 16, 51) and Nigeria (14) have demonstrated that these resources are often not available in primary care systems.

Both stakeholder groups perceived the KSHI programme as an important facilitator for implementing high-quality hypertension care because it covered costs of care for patients and provided essential resources and incentives to clinics: guidelines, staff training, medications, and diagnostic equipment. However, several barriers were still perceived to be present, including: high staff workload, discordance between healthcare provider and insurer on how health insurance and provider payment methods work, administrative challenges, and the absence of tools for tailored patient education at healthcare centre.

Both stakeholder groups perceived the shortage of qualified health workers as one of the major barriers to the delivery of high-quality hypertension care in their area. They noted that, as more people in the region had enrolled in the insurance, the patient load and the workload of the staff in participating clinics had significantly increased. In addition, healthcare staff pointed out that hypertension management is a time-intensive activity in the local context. In particular, from the perspective of doctors, more frequent consultations than recommended by international guidelines (41) are needed and more intensive patient education to ensure treatment adherence among their patient population. Suggested strategies for reducing the workload of doctors in the clinic included the redistribution of care activities from physicians to nurses and lower-level health workers (task-shifting) and the development of a benefits programme to retain experienced staff in the region. In line with suggestions from our respondents, task-shifting is generally perceived as a viable practice model for tackling workforce challenges in chronic care delivery, both in low- and high-income countries (17, 51–55). A 2014 systematic review of controlled studies from LMICs (55) reported evidence that task-shifting approaches can improve hypertension treatment outcomes. Although information on potentially effective strategies to retain health workers in rural areas in LMICs is available (56), it has been noted that such strategies cannot be developed and financed by healthcare providers alone and require commitment and investments from government (56). A recent Nigerian study suggests that the decentralisation of health systems in the country offers new and feasible opportunities for developing health worker–retention programmes at the community level (57). However, during the time of our study, concrete clinic or regional plans for addressing health worker challenges were not available.

We observed a gap between perceptions of the clinic staff on the one hand and the health insurance staff on the other hand as to how the health insurance financing and provider payment systems work. For example, whereas the provider reimbursement fees were perceived by healthcare staff as insufficient to cover all costs of care activities that are recommended by hypertension guidelines, the health insurance staff argued that the reimbursement system was designed so that the essential services for CVD prevention could be covered without increasing the co-premiums for enrolees or the financial risk to the insurance. A 2013 systematic review of the literature on provider payment methods used by CBHI schemes in developing countries (58) reported evidence that lack of consensus between providers and insurance companies about payment methods is common and can lead to a reluctance by providers to support the scheme. The review also suggested that ‘clear communication of the reasons for a particular method of provider payment can be important in garnering interest and support from providers working within the scheme’ (58). In other words, in order to promote the effective implementation of a health insurance system, in a context where such a system was previously unknown, specific attention should be given to making the different stakeholders familiar with the rationales behind and the principles of the insurance system, including the ideas about risk pooling, preventive care, and specific reimbursement arrangements. In fact, the need to establish good communication between medical and health insurance staff on issues such as provider reimbursement and claims management procedures was also emphasised by the stakeholders in our study.

Our data also show that the administrative part of the healthcare system (e.g. patient filing systems, billing and claims settlement procedures, quality assurance mechanisms) was seen as an inhibitor to high-quality hypertension care, particularly by healthcare staff. The clinic used manual paper-based records for patient registration and administrative purposes, which is common in rural SSA. Both stakeholder groups felt that the introduction of an automated patient and hospital administration system might reduce some of the perceived administrative inefficiencies and miscommunication between providers and insurers. Yet an implementation plan was not available. Currently, low-cost digital administrative systems are being developed for health systems in Africa (59). However, given the lack of experience with computer applications in rural healthcare clinics in SSA, implementing such systems will require additional personnel and financial resources for training and monitoring (60).

### Strengths and weaknesses

Qualitative interview studies with health professionals have been increasingly used to understand barriers and enablers to implementing guideline-based hypertension care in practice (28, 29, 61). Because very few of these studies have been conducted in LMICs in SSA (27–29, 61), this study expands this qualitative database with essential new information. A limitation of the study is that the findings may not be easily generalisable outside the KSHI programme, which offered some distinct possibilities to improve hypertension care for low-income patients in rural Nigeria. In other settings in SSA, additional financial or non-financial challenges to implementing high-quality hypertension care may be present. The healthcare staff participating in this study were recruited in one primary care clinic. Healthcare staff from other KSHI clinics may have raised other issues. Yet, within these limitations, we were able to include respondents from a variety of professional backgrounds, which allowed us to illuminate, by juxtaposition, perceptions of healthcare staff and insurance managers. To ensure validity of the data we conducted respondent validation (a member check) by reinterviewing 3 of the 15 respondents. We did not conduct validation exercises by having all respondents read the interview transcripts or drafts of the analysis, as this would make too great a demand on their already limited time (45). Our analytic procedures were rigorous and coding strategies and interpretations were conducted by independent researchers, whereas results were discussed in research meetings, using a reflexive team process to arrive at consensus about the most plausible codes and interpretations (45, 50). Although we used coding strategies that were designed by the sociologist Anselm L. Strauss to conduct ‘grounded’ social research (49), other qualitative methods might also have been appropriate for this study, such as qualitative content analysis (62) or thematic analysis (63). The TICD framework did not guide the first inductive stages of the data analysis but it was useful to reflect on and organise our findings at the end stage analysis. We mapped our findings on the TICD domain *incentives and resources*, because our data revealed that stakeholders perceived the availability of incentives and resources in the health system as the main condition influencing high-quality hypertension care in their own context. The domains and subdomains of the TICD framework show considerable overlap, and some of our findings could also have been coded to other TICD domains. For instance, remarks about the necessity to adapt guidelines to the local patient population could have been coded under the dimension *guideline factors*.

### Implementation and follow-up studies

By providing free access to care for patients and flexible guidelines, staff training, equipment upgrades, and care monitoring mechanisms to clinics, KSHI's quality improvement programme was designed to minimise some important factors (determinants of practice) that were perceived as potential barriers to the implementation of high-quality hypertension care by stakeholders in this study. Moreover, based on the findings from this study and a qualitative study of patient perspectives on adherence to hypertension treatment (35), our research group developed a tailored cardiovascular health education programme for patients in the region. Programmes or plans to address other factors that were perceived as challenges to the implementation of high-quality care have not (yet) been developed by local stakeholders or the research consortium (e.g. workload, provider remuneration, and administrative challenges at facilities).

This study was conducted when KSHI's CVD prevention programme had just been implemented. Follow-up studies demonstrated this programme offers a promising opportunity for financing and delivering high-quality hypertension care for low-income people in rural Nigeria. A subsequent operational cohort study (38) followed 328 hypertensive patients in a KSHI clinic for 12 months and found that the implementation of hypertension guidelines was feasible and resulted in high-quality care. For instance, after 1 year, the patient retention rate was >90%, and 67.2% had controlled BP (38). Other studies indicated that the BP of the hypertensive population in communities with access to KSHI was significantly lower compared to those without access to KSHI (34, 64). We also found that cardiovascular health education led to better medication adherence and a reduction in BP at 6-month follow-up in patients who had received guideline-based care in the context of the KSHI programme (37). However, our studies also showed that the delivery of high-quality care is very costly (65) and time-consuming (38) for providers, who are already faced with a high workload and high turnover rates of qualified personnel. This issue may threaten the sustainability of high-quality care.

### Future research

In addition to research on enablers and barriers to the implementation of CVD prevention and hypertension management in contexts in SSA where health insurance is not available, this study suggests that further work is needed to evaluate the impact of staff shortages, task-shifting, and (automated) clinic management systems on the quality and the costs of CVD prevention and hypertension care. Similarly, in collaboration with health insurance companies, more work should be done to evaluate the effect of different provider remuneration systems on the utilisation, quality, and costs of CVD prevention care in low-resource communities in SSA.

## Conclusions

In this qualitative interview study, primary care staff and health insurance managers mentioned a range of incentives and resources that need to be available to facilitate the implementation of high-quality hypertension care in rural primary care facilities in Nigeria. From the perspectives of both stakeholder groups, a health insurance programme can provide essential financial and material resources to facilitate high-quality care (e.g. guidelines, staff training, and equipment). However, other aspects also need attention (e.g. the human, material, and administrative resources that are needed for delivering quality care). Our data also illustrate that teamwork and a common understanding between providers and insurers with regard to the way health insurance operates and serves the needs of the insured population and contracted providers are important to facilitate quality care.

### Implications for practice

Health insurance is generally seen as a promising avenue towards universal health coverage and CVD prevention in SSA. In this study, qualitative interviews with healthcare staff and insurance managers provided relevant information about potential barriers to the implementation of high-quality hypertension care in the context of a CBHI programme in rural SSA and suggested entry points for strategies to address these barriers. The approach taken in this study (interviews with stakeholders) and its findings can inform the development of CVD prevention programmes in the context of other health insurance programmes in SSA.

## Supplementary Material

Enablers and barriers for implementing high-quality hypertension care in a rural primary care setting in Nigeria: perspectives of primary care staff and health insurance managers
