# Supplementary material for: Enablers and barriers for implementing high-quality hypertension care in a rural primary care setting in Nigeria: perspectives of primary care staff and health insurance managers
Source: Glob Health Action. 2016 Feb 12;9:10.3402/gha.v9.29041. doi: 10.3402/gha.v9.29041 (PMC4754020; doi:10.3402/gha.v9.29041)
Supplement: Enablers and barriers for implementing high-quality hypertension care in a rural primary care setting in Nigeria: perspectives of primary care staff and health insurance managers [file GHA-9-29041-s001.pdf]

Additional files

1. Description of the Kwara State Health Insurance (KSHI) Program
2. Example of interview guide (Topic list)
3. Example of matrix generated from inductive analysis

1. The Kwara State Health Insurance (KSHI) program (previously Hygeia Community Health Care Program)

The Health Insurance Fund (HIF) program was initiated by the international development organization PharmAccess. The PharmAccess Group is committed to providing access to quality healthcare for low- and middle-income groups in several African countries through innovative financing mechanisms and improvement of the quality of healthcare[1–5]. The HIF is developing and supporting the implementation of health insurance programs, which focus on the informal sector. The aim of the HIF program is twofold: improving the demand for and access to healthcare, by introducing health insurance for individuals with low and middle incomes as well as improving the quality of care provided in these communities. The key elements on the demand side of the HIF program are the focus on organized groups in the informal sector and their dependents, for example communities of farmers; the provision of subsidized insurance premiums; co-payment of the premium by enrolees to encourage the groups to demand quality care; and voluntary enrolment. On the supply side, the key elements include capacity building, quality assurance, involvement of the private and public sector, focus on performance-based financing, and management[6].

The HIF program uses a public-private partnership model and is implemented by PharmAccess and private African Health Maintenance Organizations (HMOs) or health insurance companies. The HMOs are responsible for execution of the program and for contracting a network of healthcare providers to provide the care for the enrolees[6]. Donor money - from both international and local sources - is used to develop and set up the insurance program and to upgrade the medical and administrative capacity of the HMOs and healthcare providers contracted under the program. The payment of healthcare providers is related to their performance. The program started in Lagos and Kwara State in Nigeria in 2007 in collaboration with the HMO Hygeia

Limited under the name of Hygeia Community Health Care[6], which later changed to the Kwara State Health Insurance (KSHI) program.

Quality assurance within KSHI program

Quality and efficiency of care are monitored through independent audits of an international quality improvement and assessment body called SafeCare[3], a partnership of PharmAccess International, the American Joint Commission International, and the South-African Council for Health Services Accreditation of Southern Africa. When a healthcare provider is contracted by the HMO, a baseline assessment in the clinic is conducted by SafeCare and a quality improvement plan is formulated. The provider specific improvement plans consist of specific targets in 13 different domains including management and leadership, human resource management, patients' rights and access to care, management of information, risk management, primary healthcare services, inpatient care, operating theatre, laboratory, diagnostic imaging, medication management, facility management and support services. The improvement plans are implemented by the healthcare providers with technical and financial support from the HMO. SafeCare monitors the progress on quality improvement through annual follow-up assessments with the SafeCare Quality Standards. Examples of quality improvement interventions include implementation of treatment guidelines (for example for hypertension), training of staff in guideline-based care, upgrading of laboratory equipment and training of laboratory staff to enable basic laboratory testing, assurance of continuous essential drug supplies, adequate medical file keeping, waste management protocols and hospital infection control protocols[6].

The KSHI program in Kwara

Since 2007, the KSHI program is rolled out in 3 regions in Kwara Sate, Nigeria: Kwara North (Kwara Edu), Kwara Central (Kwara Asa), and Kwara South (Kwara Oyun). In January 2015, 85,100 people were enrolled in the KSHI program in the 3 regions in Kwara State. The details and advantages of the health insurance program were communicated by the HMO through several channels. Activities included face-to-face information sharing (through outreach activities to the communities, house-to-house visits by enrollment officers, health education and advocacy visits to community opinion leaders) and large-scale communication and marketing activities in the target communities (through billboards, comics, brochures, flyers and elaborate

announcements and information sharing on the radio). All households living in the districts in which the program is operational are eligible for enrollment. There is no pre-enrollment screening for chronic diseases[6].

Beneficiaries are enrolled individually (as opposed to household enrollment) on an annual basis and pay a co-premium of 300 Naira or approximately US$2 per person per year. Currently, individuals are responsible for about 12% of the premium. The Health Insurance Fund and the Kwara State Government subsidize the remaining part of the premium. The Kwara State Government started contributing to the premium subsidies in 2009 and its contribution has increased from 20% to 60% over the past years while it plans to eventually take over all costs of the premium subsidy. Prior to 2009, the HIF paid the greater part of the premium subsidy through a grant from the Dutch Ministry of Foreign Affairs. The co-premium ranges from 0.96% of the average annual per capita consumption for the lowest wealth quintile to 0.16% for the highest wealth quintile (data from baseline survey 2009). The scheme’s beneficiaries do not incur out-of-pocket costs for the services covered since the clinics are paid directly by the insurance scheme[6].

The HMO has contracted 25 health facilities, 4 small health posts and 2 referral clinics to provide the care for their enrollees. Most health facilities are primary and secondary care clinics that provide outpatient services and have admission capacity. Tertiary care can be provided in the 2 referral clinics in Ilorin (the Kwara State capital) if needed.

Coverage within the KSHI program in Kwara

The insurance package provides coverage for consultations, diagnostic tests, and medication for all disease categories, including hypertension and diabetes, that can be managed at a primary care level and limited coverage of secondary care services. Secondary care services provided include radiological and more complex laboratory diagnostic tests and hospital admissions for different disease categories, minor and intermediate surgery, antenatal care and delivery care, neonatal care, immunizations, annual check-ups and HIV/AIDS treatment care and support. Excluded from the program are high technology investigations (computed tomography and magnetic resonance imaging), major surgeries and complex eye surgeries, family planning commodities, treatment for substance abuse/addiction, cancer care requiring chemotherapy and

radiation therapy, provision of spectacles, contact lenses and hearing aids, dental care, intensive care treatment and dialyses[6]. Management of acute cardiovascular events such as thrombolysis for stroke or for myocardial infarction is excluded. In case of an acute cardiovascular event, admission to a hospital for supportive care is covered including for example treatment with intravenous fluids, and antibiotics for infectious complications. The healthcare provider can claim a maximum of 12 clinic visits related to hypertension per patient. Furthermore, since a large share of the payment from the insurer to the healthcare provider is paid trough capitation, providers are encouraged to prevent overutilization of services[6].

KSHI program area and population

Kwara State is located in western Nigeria and is the fourth poorest state of the country[7]. The majority of the population lives in rural areas. The program areas included in KSHI are rural, low income farming communities. The baseline survey conducted in 2009 demonstrated that Yoruba was the dominant ethnic group in the program area (67.8%). Nupe was another large ethnic group in the program area (9.9%). Islam and Christianity were the main religions, trading and farming the main occupations. The baseline survey showed that 20% of the population lived below the poverty line of US $2 (PPP adjusted) per day. Forty percent of the population completed primary school, and a further 25% completed senior secondary school. Literacy rates were 45.7% in the program area[6]. Nigeria has among the highest out-of-pocket health spending and poorest health indicators in the world[7]. Similar to the rest of the country, Kwara State has a weak health system with inadequate government funding for health, weak governance and legislation, inadequate health infrastructure and poor service quality. Kwara State is participating in the federally funded National Health Insurance Scheme (NHIS). The majority of the enrollees, however, are individuals working in the formal sector. The baseline survey in 2009 showed that less than 1% of the population in the program were enrolled in a health insurance scheme[6], this had reached 32% at the time of this study in 2010[8].

In the program area, there were few functional healthcare facilities before the start of KSHI. Most clinics were primary care clinics; some provided limited secondary care (such as surgery and inpatient care). The implementing organization of the insurance program performed an assessment in the program area of potential healthcare providers that could be contracted under

the insurance program. Most facilities were poorly maintained, essential equipment was lacking and patient numbers were low. In 2010, KSHI's program area included three public and three private clinics.

References

1. Health Insurance Fund. Health Insurance Fund Website. 2015. http://hifund.org/. Accessed October, 2015.

2. PharmAccess Foundation. PharmAccess Foundation website. http://pharmaccess.org/RunScript.asp?p=ASP\Pg0.asp. Accessed October 10, 2015.

3. SafeCare. SafeCare Website. 2015. http://www.safe-care.org/. Accessed October 8, 2015.

4. IFHA. Investment Fund for Health in Africa (IFHA) website. http://www.ifhafund.com/. Accessed March 13, 2015.

5. Medical Credit Fund. Medical Credit Fund website. http://www.medicalcreditfund.org/. Accessed March 13, 2015.

6. Hendriks ME, Wit FWNM, Akande TM, et al. Effect of Health Insurance and Facility Quality Improvement on Blood Pressure in Adults with Hypertension in Nigeria: A Population-Based Study. JAMA Intern Med. 2014;174(4):555-563. doi:10.1001/jamainternmed.2013.14458.

7. Gustafsson-Wright E, Schellekens O. Achieving Universal Health Coverage in Nigeria One State at a Time: A Public-Private Partnership Community-Based Health Insurance Model. Washington, DC; 2013. http://www.brookings.edu/~/media/research/files/papers/2013/06/achieving%20universal%20health%20coverage%20nigeria%20gustafsson%20wright/achieving%20universal%20health%20coverage%20in%20nigeria.pdf. Accessed October 8, 2014.

8. Amsterdam Institute of Global Health and Development. The impact assessment of quality care in Africa. Report 2015. Amsterdam; Amsterdam Institute of Global Health and Development: p. 37.

2. Example of the topic list used to interview healthcare professionals (doctors)

Preparation

- Greetings

- Explain the purpose of the interview and obtain audio-recorded informed consent
- Ask participant for permission to audio-record the interview
- Make and document brief assessment of socio-demographic data (age, gender, qualifications, professional background, length of professional practice, religion, Nationality)

Introduction

In sub Saharan Africa, incidence of hypertension is increasing rapidly. If not managed effectively, hypertension often leads to poor patient outcomes, poor quality of life and an increased burden of cardiovascular diseases. Yet many hypertensive patients in Africa lack affordable access to care. To improve this situation, many African countries now adopt and implement affordable health insurance programs. However, the quality of hypertension care also contributes to treatment outcomes.

For these reasons, we would like to know your views and experiences regarding the barriers and facilitators of hypertension care in your capacity as a health care professional: what is currently being done rightly; and what is wrongly done and should be implemented in better ways.

Please feel free to air your views in your own words. All you say will remain confidential. If we report on the interviews, this will only serve as feedback to improve the system; we will not use your name or any other information by which you can be identified.

We also seek your permission to audio-record the interview for ease of transcription and analysis.

Topic 1. ***Can you think of any barriers that can make it difficult to provide high-quality hypertension care?***

1.1 Generally, what in your opinion and experience are the limitations of delivering high-quality hypertension care?

1.2 In particular, what limitations have you encountered in the course of treating hypertensive patients in the ongoing insurance program in this hospital? What issues if any have you had regarding any of the following:

- Availability and functionality of equipment
- Adequacy of hospital (laboratory) investigative capacity

- Providing health education to patients
- Communication with patients during consultations
(e.g. language barrier)
- Workload / adequacy of healthcare personnel
- Motivation
- Management support
- Availability of opportunities for refresher trainings / continuous education
- Use of clinical guidelines and standard operating procedures
- Organization/division of tasks within the healthcare team.
- Relationship with Health Insurance Company

Topic 2. ***In your experience, what can help ensure the provision of (consistent) high-quality hypertension care?***

2.1 Generally, what in your opinion and experience are the enablers of delivering high-quality hypertension care?

2.2 In particular, what facilitations have you encountered in the course of treating hypertensive patients in the ongoing insurance program in this hospital? What issues if any have you had regarding any of the following:

- Availability and functionality of equipment
- Adequacy of hospital (laboratory) investigative capacity
- Providing health education to patients
- Communication with patients during consultations
(e.g. language barrier)
- Workload / adequacy of healthcare personnel
- Availability of motivational incentives
- Management support
- Availability of opportunities for refresher trainings / continuous education
- Use of clinical guidelines and standard operating procedures
- Organization/division of tasks within the healthcare team
- Relationship with Health Insurance Company

3. Example of matrix generated from inductive analysis

Category	Sub-category	Concepts
Health Insurance	Subsidized health insurance is vital for providing standardized hypertension care for low income patients. [R1,R2]	*I used to think before now that hypertension and diabetes were not common... In fact, most of our adult consultations now are due to hypertension apart from the obstetric and gynaecologic care (R2).*<br><br>*The insured patients are better compliant with visits, drugs and advices and are better controlled. This has a lot to do with the fact that barriers to access care have been removed by the insurance (R1).*
